# Micro‐morphological feature visualization, auto‐classification, and evolution quantitative analysis of tumors by using SR‐PCT

**DOI:** 10.1002/cam4.3796

**Published:** 2021-03-07

**Authors:** Gong‐Xiang Wei, Yun‐Yan Liu, Xue‐Wen Ji, Qiao‐Xin Li, Yan Xing, Yan‐Ling Xue, Hui‐Qiang Liu

**Affiliations:** ^1^ School of Physics and Optoelectronic Engineering Shandong University of Technology Zibo China; ^2^ State Key Laboratory of Pathogenesis, Prevention, Treatment of Central Asian High Incidence Diseases First Affiliated Hospital of Xinjiang Medical University Urumqi China; ^3^ Hepatobiliary Surgery First Affiliated Hospital Xinjiang Medical University Urumqi China; ^4^ Department of Pathology First Affiliated Hospital Xinjiang Medical University Urumqi China; ^5^ Imaging Center First Affiliated Hospital Xinjiang Medical University Urumqi China; ^6^ SSRF Shanghai Advanced Research Institute Chinese Academy of Sciences Shanghai China

**Keywords:** angiogenesis, liver cancer, microenvironment, pathology

## Abstract

Tissue micro‐morphological abnormalities and interrelated quantitative data can provide immediate evidences for tumorigenesis and metastasis in microenvironment. However, the multiscale three‐dimensional nondestructive pathological visualization, measurement, and quantitative analysis are still a challenging for the medical imaging and diagnosis. In this work, we employed the synchrotron‐based X‐ray phase‐contrast tomography (SR‐PCT) combined with phase‐and‐attenuation duality phase retrieval to reconstruct and extract the volumetric inner‐structural characteristics of tumors in digesting system, helpful for tumor typing and statistic calculation of different tumor specimens. On the basis of the feature set including eight types of tumor micro‐lesions presented by our SR‐PCT reconstruction with high density resolution, the AlexNet‐based deep convolutional neural network model was trained and obtained the 94.21% of average accuracy of auto‐classification for the eight types of tumors in digesting system. The micro‐pathomophological relationship of liver tumor angiogenesis and progression were revealed by quantitatively analyzing the microscopic changes of texture and grayscale features screened by a machine learning method of area under curve and principal component analysis. The results showed the specific path and clinical manifestations of tumor evolution and indicated that these progressions of tumor lesions rely on its inflammation microenvironment. Hence, this high phase‐contrast 3D pathological characteristics and automatic analysis methods exhibited excellent recognizable and classifiable for micro tumor lesions.

## Graphical abstract

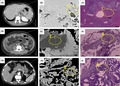

## 1 INTRODUCTION

Cancer continuously threatens human life and health globally, such as the latest clinical survey shows the 5‐year survival rate of malignant tumors in digest system is 20%–40% on average.^1^ Research progresses on diagnosis and therapy strategy of malignant tumors has been achieving worldwide in many aspects, meanwhile it faces enormous challenges due to the complexity of tumorigenesis and development.^2^ Actually, the formation of tumor stroma plays an important role in causing cancers, and the interactions between stromal cells and cancer cells is essential for tumor typing and progression.^3^, ^4^ Small morphological changes in tumor microenvironment, such as in tumor microangiogenesis and tiny tissue lesion aspects, are likely become the distinguishing feature of cancer.^5^, ^6^ Currently, the techniques of medical imaging, like positron emission tomography‐computed tomography (PET/CT), magnetic resonance imaging (MRI), ultrasound, and histopathology examination, are still the main means of solid tumor detection and evaluation. Each technique has its own usable range owing to the limited spatial resolution, applicability for soft tissue or bone, complexity of sample preparation and three‐dimensional (3D) observation, especially inaccessible 3D structural information of soft tissues at the micrometer length scale for conventional medical imaging techniques in hospital.^7^, ^8^ Some vascular geometric parameters like micro‐vessel volumetric density (MVD), vessel diameter and length, can provide prognostic significance due to the differences between tumor tissues and adjacent normal tissues.^9^, ^10^ Actually, a high‐resolution 3D nondestructive observation and quantitative analysis of soft solid tumor micro‐morphology still remains a challenging task for revealing the tumor micro lesion evolutionary mechanism, and this will be more suitable for the application of different deep‐learning algorithms to improve the auto‐classification accuracy of tumor diagnosis, rapidly developed with widely using high‐performance computer.^11^, ^12^ In this work, we employed the synchrotron radiation X‐ray phase contrast microtomography (SR‐PCT) and phase retrieval algorithm (PR), superior in signal‐to‐noise ratio and density, to visualize micro‐tissue tumor features and its irregular angiogenesis networks.^13^, ^14^, ^15^ It is necessary for quantitative evaluation of tumor lesion and its antiangiogenic drugs to identify malignant tumorigenesis, feature typing and staging, immature vessel structures, and tumor progression evolution path. However, the 3D observation and measurement of micro‐morphological features, less than 200 μm in diameter, is still limited or not satisfying. This affects the better understanding of mechanism of tumor evolution.^16^ We need to take more efforts to study micro‐morphological characteristics and biology of tumor lesions. First, the high phase‐contrast tomographic images of specimens of post‐operation or biopsy via SR‐PCT experiment and PR was obtained and compared with other modality images in a trans‐scale correlation way. Second, the multiscale 3D pathological images with diagnosis significance were displayed and the statistical calculation of the volumetric tumor angiogenesis network was achieved to quantitatively evaluate the tumor lesions. Third, the deep convolutional neural network (DCNN) was successfully employed in tumor feature recognition and auto‐classification, and consists of four different layers, namely convolutional, activation, pooling, and fully connected layers.^17^ In this study, the tumor micro‐morphological features with high density resolution can be highlighted and extracted for the AlexNet based DCNN learning model,^18^ characterized with the simple and efficient cascaded stage for training and optimizing. For a large enough training dataset of the AlexNet‐based DCNN, the image enhancement and rotation were applied to produce the fourfold augmented dataset of 0°, 90°, 180°, 270° for the automated classification of tumor images, and 49152 images finally for each tumor specimen. Finally, we extracted the tumor texture and grayscale characteristics of the gray level co‐occurrence matrix (GLCM), gray‐gradient co‐occurrence matrix (GGCM), gray level histogram (GH), gray level differential statistics (GDS), and combined both feature screening methods of area under curve (AUC) and principal component analysis (PCA) to eliminated the redundant feature quantity.^19^, ^20^, ^21^ Therefore, the correlation and staging of tumor microscopic structural changes was quantitatively analyzed and indicated that the tumor microvasculature growth has close relationship with the inflammation of tumor microenvironment.

## 2 MATERIALS AND METHODS

### 2.1 Tumor specimens and experimental preparation

The postoperative tumor specimens used for this study were obtained from the first affiliated hospital of Xinjiang Medical University (XJMU), with the consent of the patients and their families and approved by the ethics committee of XJMU. All tumor specimens of 100 cases, including liver, intestine, and stomach, showed moderate differentiation and low differentiation based on pathological results. Without the preoperative use of contrast agent, all tumor tissues were washed by phosphate buffered saline and fixed by 10% neutral formalin in postoperative 30 min. Prior to imaging experiments, all formalin‐fixated specimens were cut into cylinder experimental samples with diameter of 10, 6, 1 mm for adapting to different imaging field of views (FOVs) with different resolution of 6.5 μm/pixel, 3.25, 0.65 μm/pixel. A process of graded dehydration series of ethanol solution, including 40% ethanol solution (4°C) and 50%, 70%, 80%, 90%, 95%, and 100% ethanol solution (indoor temperature), were employed for all cut samples, then fully dried and stabilized in a plastic tube to avoid the motion artifacts associated with fresh tissue deformation and degeneration or movement during the brilliant synchrotron X‐ray imaging measurement. After the SR‐PCT experiments, all tumor samples were preserved in 10% PFA solution for subsequent histopathological examination.

### 2.2 Examination of histopathology

All experimental samples were processed through dehydration and paraffin embedding, and then cut continuously into 5 μm slices with subsequent hematoxylin and eosin (H&E) staining for LEICA‐DM3000 microscopic observation.

### 2.3 Phase Contrast microtomography (PCT)

**FIGURE 1:**
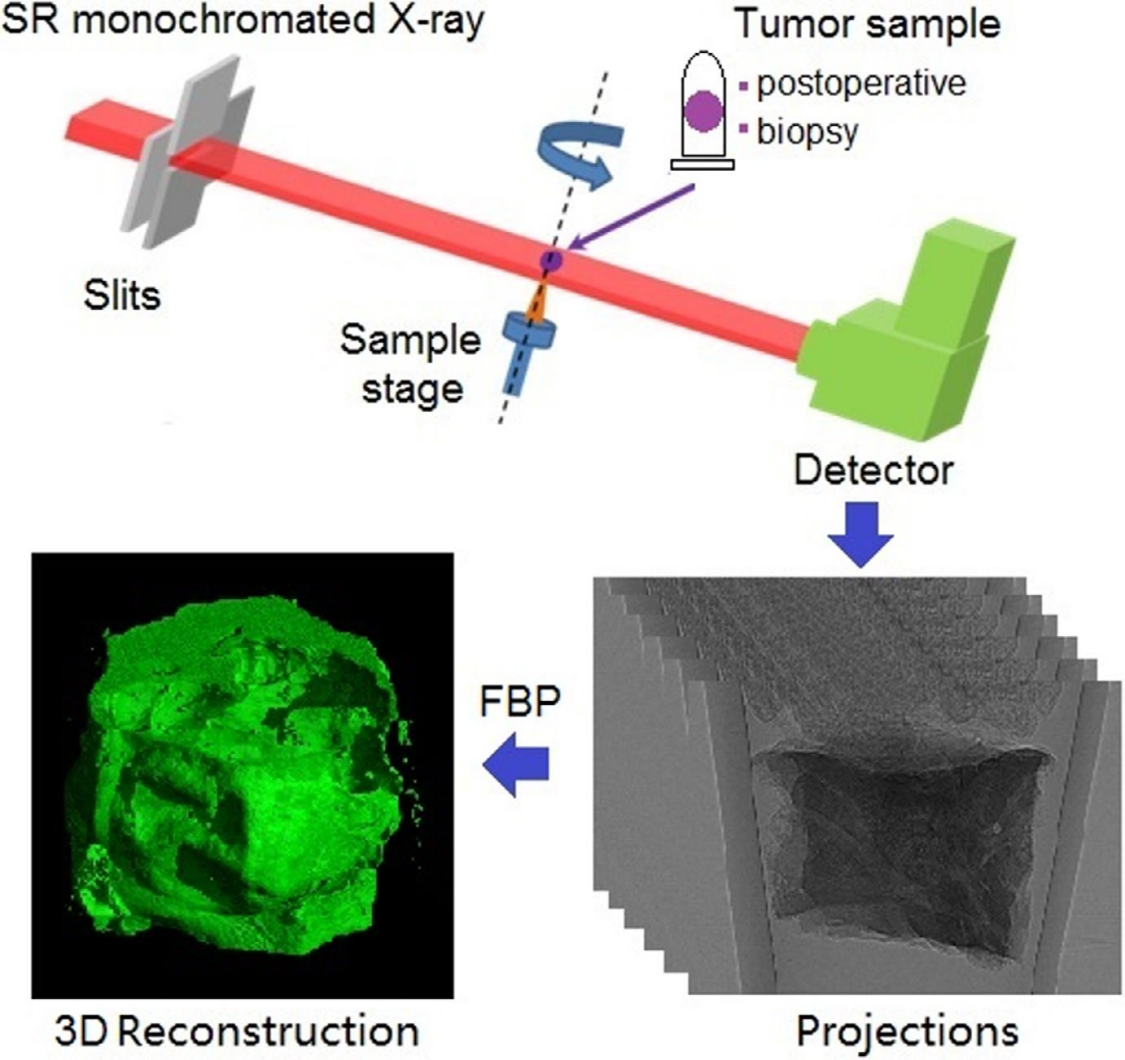
Schmatic of SR‐PCT experimental setup and biomedical 3D reconstruction. SR‐PCT, synchrotron‐based X‐ray phase‐contrast tomography

The SR‐PCT experiment was performed in x‐ray imaging and biomedical BeamLine BL13W1, at Shanghai Synchrotron Radiation Facility (SSRF). The high‐brilliance Synchrotron beams produced from the 3.5GeV storage ring, can be monochromated through a double‐crystal monochromator to select X‐ray photon energy of 15 keV, and the sample stage is located at the 34 m downstream of the source approximately with a monochromatic X‐ray beam of 50 mm (H) × 4 mm (V). There is not any optical element between sample and detector in our SR‐PCT experimental setup, shown in Figure 1, consisting of a six‐dimensional high precision sample stage. The CMOS detector (HAMAMATSU C11440‐22C) with pixel format of 2048*2048 and original pixel size of 6.5 μm was coupled with 25 μm‐thickness scintillator and camera lens assembly, converting the incident X‐rays into visible light and focused onto the CMOS device through a lens system of the magnification factor of 1×, 2×, 10×. The FOVs are 13, 6.5, 1.3 mm^2^, respectively, corresponding to different effective pixel sizes 6.5, 3.25, 0.65 μm. The corresponding spatial resolution is about twice as the effective pixel size. Actually, the synchrotron‐based imaging techniques are generally used for studying high‐resolution ex vivo specimens, not for patients, mainly due to the limitation of biosafety doses. For whole organs or small animals, there will some medium and long beam lines with large size beam spots and equipped with large size detectors.

### 2.4 PR and image processing

A raw SR‐PCT projections was acquired at sample‐to‐detector distance (SDD = 7 cm). The SDD yields a Fresnel‐diffraction projection pattern on the detector plane, depicted as projections in Figure 1 and characterized as weak‐absorption contrast and edge enhancements at the boundaries and interfaces induced by phase distortion. For hard X‐ray beams, the propagation‐based phase‐sensitive imaging can simply and directly achieve phase contrast, by placing a detector at a SDD downstream of a sample without any additional X‐ray optics. The reconstructed intensity distribution of SR‐PCT contains the 3D map of the linear attenuation coefficients and the 3D map of Laplacian of refraction index decrements of a sample. In the direct phase contrast imaging, it is difficult to achieve adequate image contrast between those bulk areas of weak‐absorption samples, except for the edge enhancements at the boundaries and interfaces arisen from the phase distortion. Therefore, a complicated PR process is required for visualization and quantitative measurement of soft biomedical tissues.

All projection data was processed via background correction prior to PR, was applied to retrieve a phase map wit high density resolution from only a single free‐space propagation radiography. For obtaining an enhanced image contrast of weakly absorbing samples in hard X‐ray domain, the PR was employed into extracting a high phase‐contrast map from any two‐dimensional projection at a given single‐distance *D* as following:

(1) $$\varphi \left(x,\;y\right)=\frac{\delta }{2\beta }\cdot \ln\left\{F^{‐1}\left[\frac{F\left(I_{D}\left(x,\;y\right)/I_{\text{in}}\right)}{\left[\cos\left(\pi \lambda D[u^{2}+v^{2}]\right)+\left(\delta /\beta +\pi \lambda D[u^{2}+v^{2}]\right)\left(\sin\left(\pi \lambda D[u^{2}+v^{2}]\right)\right)\right]}\right]\right\},$$

where $\lambda =8.27\times 10^{‐11}m$ is the X‐ray wavelength, *D* is the SDD. (*x*, *y*) and (*u*, *v*) denote the Cartesian coordinate and the Fourier conjugate coordinate, F and $F^{‐1}$ denote the forward and backward Fourier transform operators, respectively. $I_{D}\left(x,y\right)$ is the transmitted X‐ray intensity, $I_{\mathrm{in}}$ is the incident intensity.

ε=δ/β is the ratio of real and imaginary parts (*δ* and *β*) of complex refraction index, representing the sensitivity of SR‐PCT. For a constant ε of soft biomedical samples, it can be estimated from X‐ray databases, for instance CXRO, 2012, NIST database, or Henke et al. (1993).^22^ The tomographic reconstruction based on standard filtered back‐projection algorithm by using the software of PITRE3 compiled by BL13W1. The 3D quantitative segmentation and rendering was achieved through the scientific and industrial visualization & analysis software of Amira 6.0 (Visage Imaging) at SSRF.

### 2.5 Statistical analysis

The statistical analysis in this study was conducted with the SPSS Statistics (version 25; IBM). All results are showed as mean ± SD and *t*‐test was performed for comparison between data sets conforming to normal distribution, where a *p*‐value lower than 0.05 was predetermined as the significant level for statistical analysis.

### 2.6 DCNN learning and classification

**FIGURE 2:**
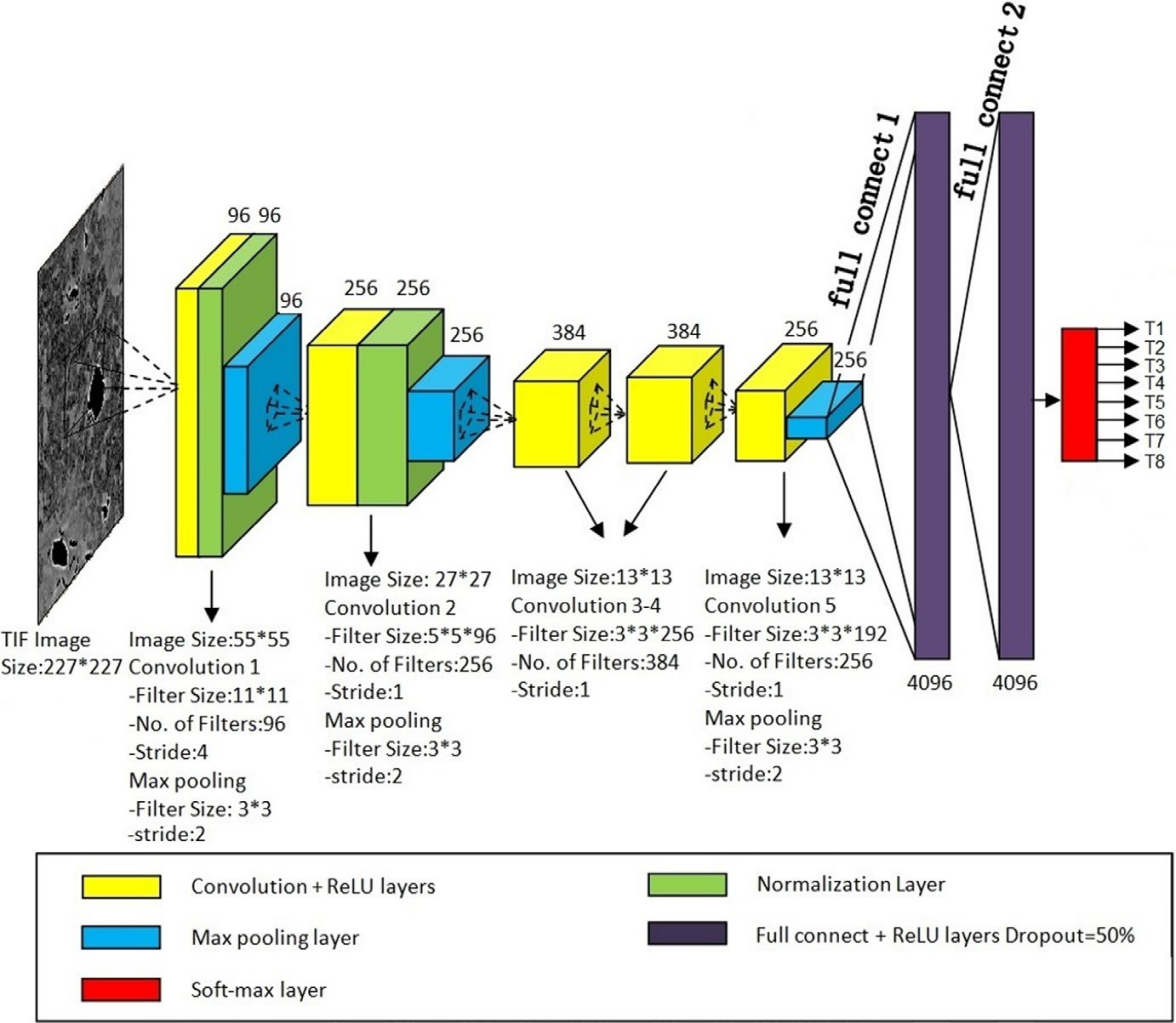
Schematic diagram of the DCNN for tumor micro‐classification based on SR‐PCT. DCNN, deep convolutional neural network; SR‐PCT, synchrotron‐based X‐ray phase‐contrast tomography

The DCNNs architecture is a biologically inspired class of deep learning models that has achieved excellent performance on visual and speech recognition problems. A typical DCNN involves four types of layers: convolutional, activation, pooling, and fully connected layers. Our DCNN model was constructed based on a very famous DCNN named AlexNet, which was first proposed by Alex Krizhevsky et al. In the 2012 ImageNet Large Scale Visual Recognition Challenge (ILSVRC‐2012). Compared with the other structure‐complex DCNN architectures (e.g., GoogLeNet, VGG et al.),^23^, ^24^ AlexNet is a structure‐simple and high efficient DCNN, which is easy to train and optimize. In this study, the AlexNet‐based DCNN was employed to perform the automated classification of eight types of tumor lesions (T1–T8, depicted in next section), extracted, and generalized from image characteristics with the resolution of 6.5 μm/pixel in our SR‐PCT experiments. The architecture generally consists of convolutional (yellow cube), activation (green cube), pooling (blue cube), and fully connected layers (purple cube) followed by rectified linear unit ReLU and characterized with the simple and efficient cascaded stage for training and optimization,^25^ as shown in Figure 2. Each original tomographic dataset of our tumor specimens included transverse, saggittal, coronal section images, cropped into 1024*1024 pixels and divided into four subgraphs with 512*512 pixels, then resized into 227*227 pixels for an input image, 12288 original images per specimen, 100 tested specimens in total. The output results denoted the probabilities of eight types (T1–T8) of tumor lesions, calculated by a soft‐max function (red cube). In order to decrease over‐fitting in neural networks through avoiding complex co‐adaptations on training dataset, the dropout rate in our networks was 50%. Our DCNN platform was assembled by the Caffe package based on the Ubuntu 16.04 operation system, which consumed about 10 h for training the models by using two graphic cards (Nvidia GeForce GTX TITAN X, 12GB), an Intel(R) i7‐4790 K CPU @4.00 GHz and the RAM of 32 GB.

## 3 RESULTS

### 3.1 Morphological comparison and correlation

**FIGURE 3:**
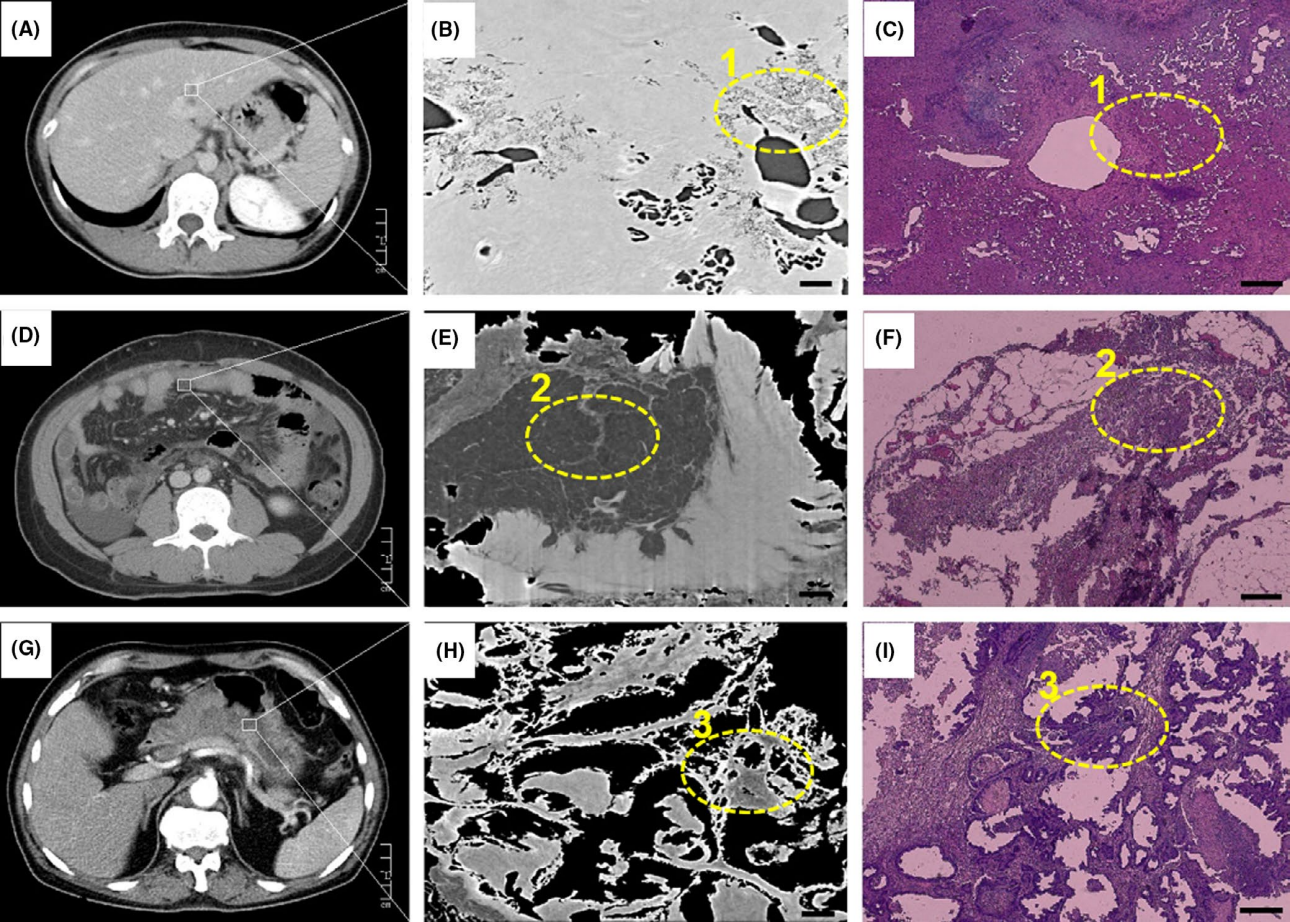
Sectional images of liver (A–C), intestine (D–F), and stomach (G–I) cancers, respectively obtained by conventional CT (A, D, G; scalebar = 1 cm ), SR‐PCT (B, E, H; scalebar = 100 μm), Histopathology (C, F, I; scalebar = 100 μm). Lesions in (B, E, H) corresponding to the white rectangle regions in (A, D, G), and the similar lesions between different imaging modalities highlighted by yellow circle 1,2,3. SR‐PCT, synchrotron‐based X‐ray phase‐contrast tomography

Synchrotron‐based tans‐scale sectional images of three types of tumor specimen showed more detailed structures and morphological characteristics than that of conventional CT in hospital, and were well matched with those of pathologic examination (H&E), as shown in Figure 3. The macro‐ and advanced tumor lesions were well diagnosed as abnormal gray spots or areas by conventional CT in Figure 3A,D,G. The micro‐pathologic features, such as those morphologies of arterioles, venules, sacs, internal septa, and inflammation regions in soft tumor tissues, can be clearly observed and demarcated from the normal tissues at the level of microns by the SR‐PCT technique, shown in Figure 3B,E,H corresponding to white rectangle areas. The micro‐pathologic inner‐structural information can subtly describe tumorigenesis and metastasis, associated with tumor classification and neoplasm staging precisely. According to the comparison labelled by the yellow circles in the Figure 3C,F,I, the SR‐PCT slices were well matched with those of H&E microscopic examination, which demonstrated the validity of tumor evaluation. Furthermore, the SR‐PCT technique can provide the three 3D structural observations without complex staining processes, exhibiting the soft tissue density resolution of different tumor lesions in digest system.

### 3.2 Multiscale 3D micro‐pathological visualization of SR‐PCT

**FIGURE 4:**
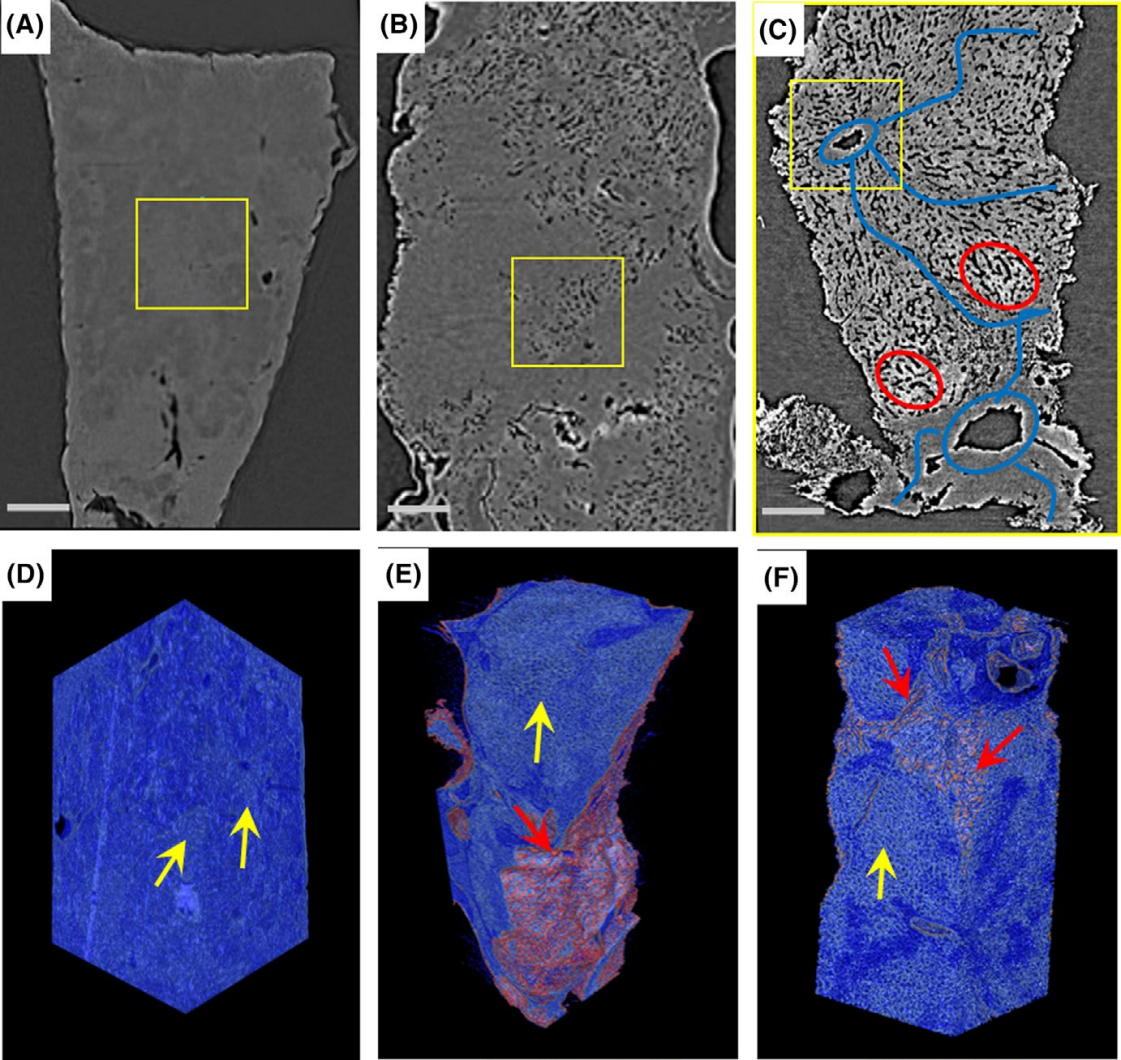
Tomographic sections and their 3D renderings of typical liver tumor specimens with different effective pixels: 6.5 μm/pixel (A, D; scalebar: 500 μm), 3.25 μm/pixel (B, E; scalebar: 250 μm), 0.65 μm/pixel (C, F; scalebar: 50 μm). The enlarged views clearly display different pathological micro‐morphologies by comparisons between yellow rectangles

The SR‐PCT‐based nondestructive 3D multiscale reconstruction exhibited comprehensive and volumetric observation of tumor lesion, helpful for more insightful assessment of tumor evolution. The reconstructed results with different resolutions (6.5, 3.25, 0.65 μm/pixel, seen in Figure 4A–C) showed the imperceptible and tans‐scale changes, such as hepatic sinusoid, perivascular inflammation, microvascular proliferation, etc. Especially, the hepatic fibrosis stemming from hepatic veins and hepatic microcalcification can be identified and depicted by blue lines and red circle respectively in the higher resolution, shown in Figure 4C. In particular, the 3D renderings of peripheral normal microtissues (blue), tumor inflammation (yellow arrows), microcystic infiltration (red arrows), labeled in Figure 4D–F, enable the 3D trans‐scale visualization and correlation representation of physiology and pathology. It is impossible for conventional histopathologic examination to provide such 3D nondestructive inner micro‐visualization.

### 3.3 Microvessel network in liver tumors

**FIGURE 5:**
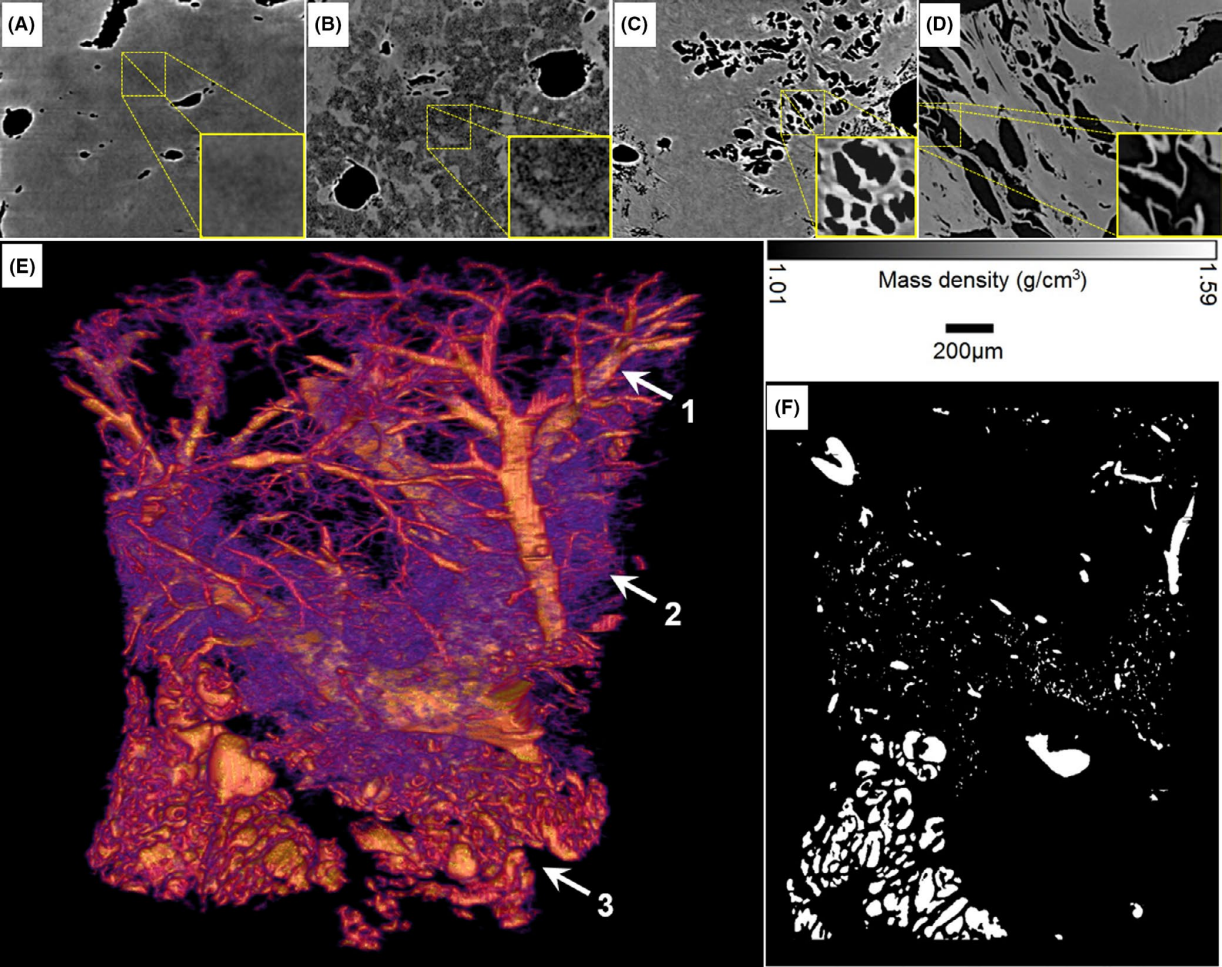
Visualization of evolutionary process and quantitative analysis of the liver tumor microvessels. Sectional images with the mass density scale‐bar of (A) inflammatory infiltration, (B) angiogenic micro‐cyst cluster, (C) micro‐vascularization of tumor, (D) abnormal microvessel network in liver tumor microenvironment. The 3D rendering of a tumor vessel network (E), arrow denoted dendritic vessels, arrow 2 microvascular cystic proliferation, arrow 3 abnormal blood supply network and their corresponding binary segmentation (F)

Tumor angiogenesis is an important hallmark of cancer in early diagnosis and treatment assessment. In this SR‐PCT experiment, the evolutionary process of liver tumor angiogenesis was revealed and generalized in Figure 5, which underwent the inflammatory infiltration (a), disseminated angiogenic micro‐cyst clusters (b), microvascularization of tumor (c), abnormal microvessel network (d). The 3D tumor vessel network is characterized by highly irregular morphological development, including the dendritic vessels (arrow 1), microvascular cystic proliferation (arrow 2), abnormal blood supply network (arrow 3) as seen in Figure 5E. The visualization of tumor microenvironment is essential to study on tumor formation and metastasis. For quantitative analysis of tumor vascular specificity, the binary segmentation based on Otsu algorithm^26^ was achieved and shown in Figure 5F, which is generally necessary for the statistical analysis of geometric quantitative parameters, represented in discussion section.

### 3.4 Auto‐classification of tumor lesions

**FIGURE 6:**
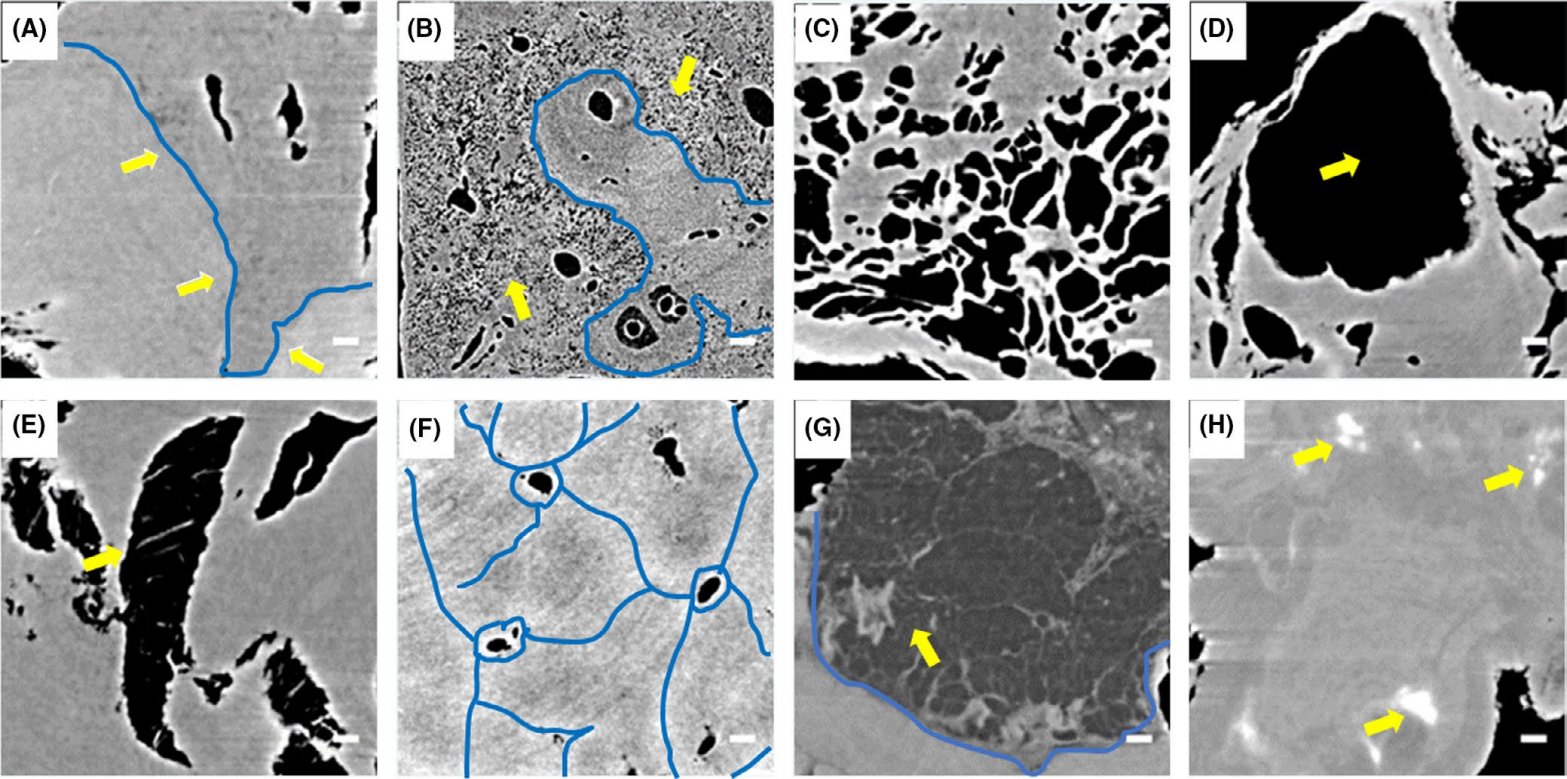
Auto‐classification standard set consisting of eight characteristic types of tumor growths in digest system based on the synchrotron radiation micro‐computed tomography (SR‐μCT) technique. (A) T1: disseminated inflammation, demarcated by blue line and the normal area shown with arrows; (B) T2: circumvascular micro‐cyst cluster, demarcated by blue line and shown with arrows; (C) T3: micro‐vascularization dilation; (D) T4: cavitation lesions, denoted by the yellow arrow; (E) T5: abnormal vessel network with septa; (F) T6: multifocal fibrosclerosis, indicated by blue lines; (G) T7: radiolucent rings and low‐density mass, shown clearly by the blue line and arrow; (H) T8: microcalcification, indicated by arrows; T1–T6 high incidence in liver cancer, T7 in intestinal cancer, T8 in gastric cancer. The length of scale bar is 150 μm

There were three pathologists of the first affiliated hospital of XJMU were responsible for feature extraction and labeling. All 3D image data of samples derived from our SR‐PCT with high phase contrast and high spatial resolution for DCNN training and testing. For conventional CT, it is difficult to identify the fine structures of soft tissue tumors during early or middle stages, which hardly implements the typing and staging for malignant tumor diagnosis. However, the high phase‐contrast microstructures reconstructed from SR‐PCT facilitates the auto‐classification based on the high density and spatial resolution of 3D microstructures. The typical feature sets consisting of eight types of soft tissue tumors in digest system were presented and depicted respectively in Figure 6, which plays an important role in the auto‐classification training via using the AlexNet‐based DCNN model.

The auto‐classification model of tumor lesions was trained and tested by the volume of 1,228,800 image datasets, including three sets of training (70%), validation (15%) and test (15%). The tumor features were extracted and auto‐classified into eight types (T1–T8). The accuracy of classification model can be calculated by the threefold cross‐validation and the random number of image dataset listed in Table 1. The statistic results showed the classified accuracies based on SR‐PCT sectional images all reached more than 90%, and average accuracy of eight types 94.21%, exhibiting a high sensitivity for phase‐contrast tumor structural recognition. The accuracy of T4 and T5 are lower than average accuracy due to their higher morphological similarity compared to other groups.

**TABLE 1:** the classification accuracies for SR‐PCT‐based model by 3‐fold cross‐validation

Types of tumor lesions	Statistic results			
	Average accuracy (%)	Dataset_1	Dataset_2	Dataset_3
T1	93.82	51157	51396	50962
T2	96.33	52018	52307	51952
T3	95.51	50983	50365	50931
T4	90.35	51201	51863	52011
T5	91.40	52008	52034	51209
T6	95.65	50991	51123	50978
T7	95.63	51564	51200	51089
T8	94.98	50872	50239	51200

## 4 DISCUSSION

**FIGURE 7:**
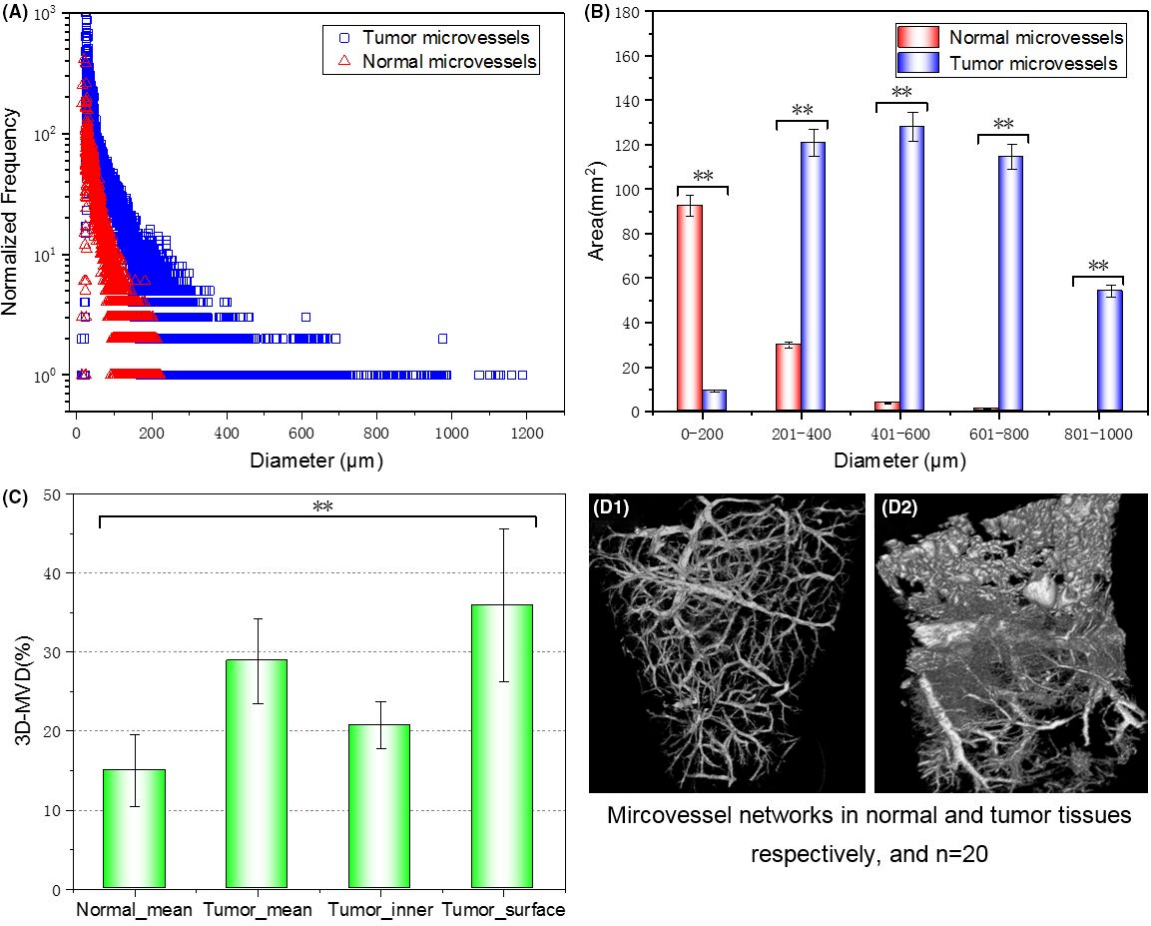
Quantitative analysis and comparisons of microvessel network of tumor liver based on the SR‐PCT technique. The distributions of different diameters of microvessels in liver tumors and normal tissues (A); The total sectional areas in different diameter intervals (B); The statistical results of 3D‐MVD in different liver tumor regions (C); The typical statistical sample groups of microvessel network in normal liver tissues (D1) and liver tumors (D2) respectively, and each sample set consisting of 20 samples; ** denoting *p* < 0.05. SR‐PCT, synchrotron‐based X‐ray phase‐contrast tomography

Conventional CT currently plays a critical role in medical imaging for tumor diagnosis and therapeutic evaluation. It is well‐known that early detection of malignant tumors is essential to improving survival. However, these fine structures and variations associated with early malignant tumors of soft tissues are often unable to be detected with conventional attenuation‐based CT due to small density change in soft tissue's tumor, limited absorption resolution and insufficient X‐ray source brightness. Although the histopathology examination can provide high‐resolution slices, it is necessary to undergo complex preprocess of samples, including dehydration, paraffin embedding, cutting, staining, baking, etc. In addition, it cannot satisfy the 3D and nondestructive observation, and is not sufficient in diagnosing and understanding of carcinogenesis from volumetric fine‐structural features and 3D perspectives. However, the SR‐PCT combined with PR exhibited the highly phase‐sensitive and multiresolution 3D visualization of fine structural characteristics and their micro‐morphological types (T1‐T8) of malignant tumors in our experiment, helpful to reduce uncertainty of early diagnosis and avoid overuse of biopsy, percutaneous needle aspiration, or surgical resections. In particular, the SR‐PCT can guarantee feature segmentation and structural parameter statistics in tumor microenvironment due to its high contrast‐to‐noise ratio, demonstrated by the quantitative analysis of microvessel network of the liver tumors, shown in Figure 7, including the distribution of diameters, sectional areas, volumetric densities (3D‐MVD) of liver tumor microvessels, and their comparisons with that of normal liver tissues. Each statistics was implemented in sample volume of 3.5 mm (height)*3.5 mm (width)*3.5 mm (length), and the sample size in every statistical group is 20 (*n* = 20). It is evident that the size distributions of liver tumor microvessels are significantly wider than that of normal tissues, and the degree of tumor lesions is increasing with rapid tumor vessel growths. This often provides prognostic significance from immature vessel structures to abnormal blood flow. The vascular diameter of normal tissue changes proportionally with vessel branching evenly, but that of tumor vasculature will not arborize in order and their diameters become thicker as tumor‐vessel tortuous extension. The two‐dimensional microvessel density in histopathology is critical for tumor clinical diagnosis, and the volumetric microvessel density (3D‐MVD) in this study was calculated in different tumor tissue regions (inner regions and surface regions) and compared with adjacent normal tissues. From the Figure 7C, we can see that the 3D‐MVD results vary remarkably and the average of tumor 3D‐MVD is obviously higher than that of normal tissue, and furthermore the blood flow in tumor surface areas higher and complicated than that in tumor inner areas and normal tissues due to the irregular vascular structure and density distribution. The findings are helpful for further study on the irregular dynamics in tumor blood supply associated with abnormal 3D‐MVD and their topological structure.

To further investigate the malignant tumor early diagnosis, it is necessary to explore the screening and classification method at the level of microscopic structures of tumor lesions. Here we trained the AlexNet‐based DCNN model by using the SR‐PCT datasets to identify eight types of tumor lesions in digesting system, which achieved the high accuracy of auto‐classification (average 94.21%) and demonstrated the excellent model performance. This is mainly because the SR‐PCT combined with PR can provide the high contrast and resolution sectional images for discriminations of the fine structural characteristics and morphological distributions with small tissue density changes. The AlexNet‐based DCNN model with appropriate 5 convolutional layers facilitate the high auto‐classification accuracy based on the SR‐PCT‐based training model, which can be served as a complementary tool for conventional CT‐based training application. Due to the pathological features of SR‐PCT images at the resolution level of about a few microns, they are totally different from that of conventional CT and histopathological images, and our image data can provide more abundant tumor micro‐morphological features, and there are few reports on DCNN of SR‐PCT experimental data. It may be unfair to compare the accuracy of DCNN based on different modalities and scales imaging datasets. The accuracy of DCNN classification model trained by HE pathological sections was 78.2% for four types, reported in our previous study.^18^ However, the average classification accuracy of SR‐PCT data can reached up to about 94.2% for eight types, benefiting from the phase‐contrast micro‐morphologcial features.

For quantitative analysis of the micro‐morphological correlation of inflammatory microenvironment and malignant liver tumor growth and metastasis, we extracted the 49 texture features of tumor SR‐PCT sectional images using GLCM, GGCM, GH, GDS, wavelet, respectively, listed in Table 2.

**TABLE 2:** Feature extraction for tumor micro‐structural quantitative analysis

Feature types	Eigenvalue	
GLCM	(0°, 1)	Energy (A1), homogeneity (A2), contrast (A3), correlation (A4)
	(45°, 1)	Energy (B1), homogeneity (B2), contrast (B3), correlation (B4)
	(90°, 1)	Energy (C1), homogeneity (C2), contrast (C3), correlation (C4)
	(135°, 1)	Energy (D1), homogeneity (D2), contrast (D3), correlation (D4)
GGCM	Small gradient dominance (E1), large gradient dominance (E2), uneven distribution of gray scale (E3), uneven distribution of gradient (E4), energy (E5), mean value of gray scale (E6), mean value of gradient (E7), mean squared error of gray scale (E8), mean squared error of gradient (E9), correlation (E10), gray entropy (E11), gradient entropy (E12), mixed entropy (E13), inertia (E14), inverse difference moment (E15)	
GH	Mean value (H1), Variance (H2), Skewness (H3), Kurtosis (H4), Energy (H5)	
GDS	Contrast (I1), Angular second moment (I2), Entropy (I3), Mean value (I4)	
Wavelet	M1, M2, M3, M4, M5, M6, M7, M8, M9	

Abbreviations: GDS, gray level differential statistics; GGCM, gray‐gradient co‐occurrence matrix; GH, gray level histogram; GLCM, gray level co‐occurrence matrix.

**FIGURE 8:**
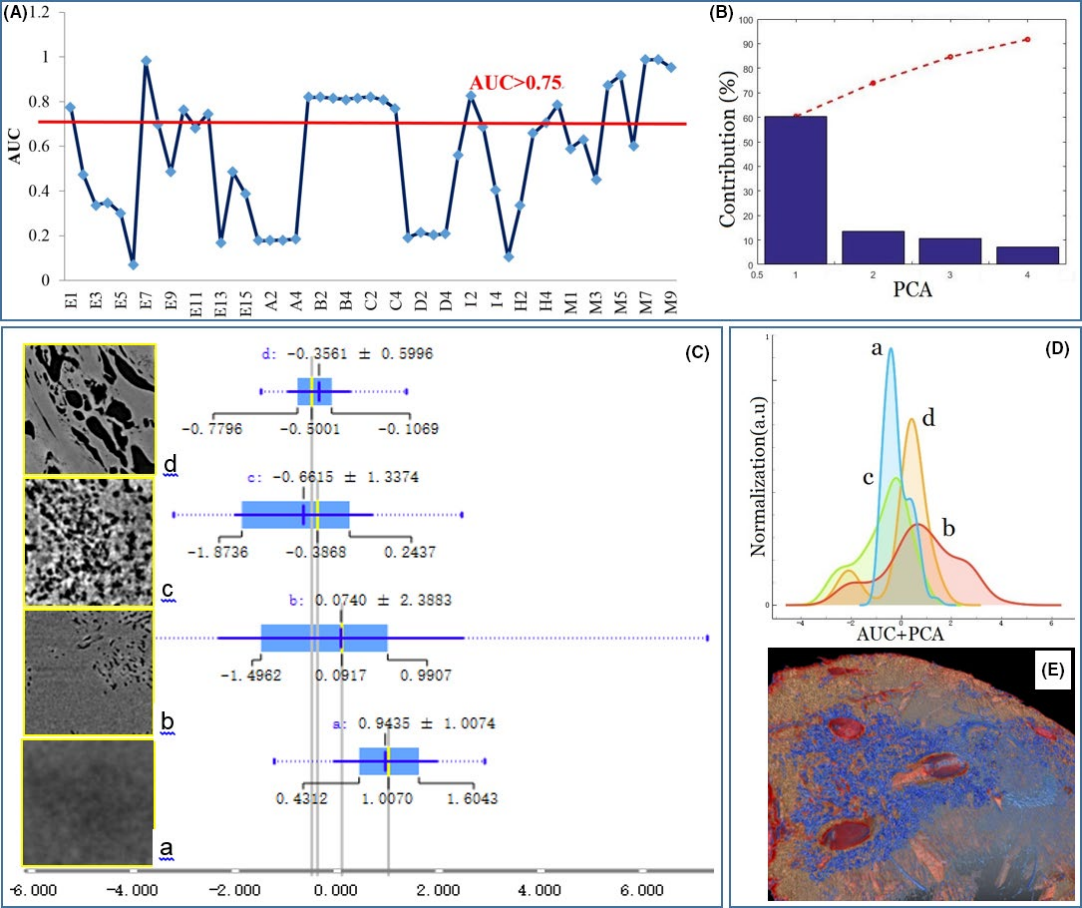
Quantitative analysis of micro‐morphological development in the liver tumor growth and metastasis. (A) Feature selection based on the AUC (area under curve) method from 49 features, there are 18 AUCs of features greater than 0.75; (B) four PCAs (principal component analysis) whose accumulating contribution rate greater than 90%. The optimal combination of features based on AUC and PCA methods can be served as quantitative analysis of different liver tumor structural lesions; (C) four typical microscopic pathological SR‐PCT features in liver tumor lesions, and their main PCAs value distribution; (D) The normalization connections and changes of four typical lesions in liver tumor microenvironment; (E) the 3D rendering of liver tumor micro‐morphological climacteric from inflammation to vascularization. SR‐PCT, synchrotron‐based X‐ray phase‐contrast tomography

In order to eliminate the correlation and redundancy effects between the 49 features, we combined AUC of ROC and PCA to optimize the feature selection and analysis. The screening criteria were set as greater than 0.75 by calculating the AUCs of the features, and there are 18 adequate features shown in Figure 8A. Then the screened AUC feature set was further reduced dimensionally by the PCA method, in which the eigenvalue is greater than 1, and the accumulating contribution rate greater than 90%. There are 4 PCAs that meet above criteria, including PCA1_60.34%, PCA2_13.59%, PCA3_10.69%, and PCA4_ 7.10%, shown in Figure 8B. The mean ± SD of PCAs (PCA1 + PCA2 + PCA3 + PCA4) of the four typical tumor microfeatures were calculated and shown in Figure 8C, indicated that the mean PACs of tumor structural features successively distributed in the range of ±2, and shifted from positive value to negative value with the deterioration of tumor lesions, and stage c displaying tumor microvascularization dilation has the inflection point of PCAs shifting. In Figure 8D, the curve of stage a has a high and narrow peak denoting the disseminated inflammation stage, the curve of stage b has a wide and flat peak denoting circumvascular micro‐cyst cluster, the curve of stage c became narrowing gradually and appeared differentiation denoting the tumor microvascularization dilation and small tissue lesion, and the curve of stage d took on distinct differentiation and arise two lesion peaks denoting the tumor abnormal microvessel network and severe tissue lesions in liver tumor microenvironment. Actually, the tumor microvascularization dilation of stage c is an important demarcation of tumor lesion mutation, rapidly developing in tissue inflammation microenvironment, shown in Figure 8E. Therefore, the micro‐morphological lesions of tumor based on SR‐PCT technique can be quantitatively measured and assessed by machine learning, which will provide data support for discrimination of benign or malignant tumor, and tumorous grading and staging.

In addition, the synchrotron‐based phase‐contrast computed tomography can achieve the fast, low‐dose, 3D imaging for soft biomedical samples for hard X‐rays. In our experiment, the exposure times are 5, 20, and500 ms, corresponding to different effective pixel sizes of 6.5, 3.25, and 0.65 μm, and projection numbers 1080, 720, and 600, respectively. The achievable doses can be approximately calculated as following:

$$\text{Dose}=F_{x}\cdot E\cdot \frac{\mu_{\text{en}}}{\rho },$$

where *E* is the photon energy, *F_x_* is the total photon fluence per tomographic scan (3.0*10^10^photons/mm^2^/s @15 keV), and *μ*_en_ */ρ* denotes the mass energy‐absorption coefficient of samples. The estimated doses are in the range of 5000‐10,000 Gy per tomographic scan and approximate resolutions between 2 and 13 μm, producing images with an appropriate level of signal‐to‐noise ratio. Actually, there were no artifacts found in our reconstructed results due to the experimental samples preprocessed with dehydration and fixation. In addition, the sample temperature didn't rise much because most of hard X‐ray photons transmitted rather than absorbed for soft tissues.

## 5 CONCLUSION

The high phase‐contrast trans‐scale 3D micro‐morphological characteristics of tumors reconstructed from SR‐PCT technique is better helpful for understanding of tumorigenesis and microvascular abnormalities, which is also a complementary tool for diagnosis of early tumor screening and typing. The findings of multimodality and multiscale correlations and complementarities in tumor microenvironment will be essential for determining the early diagnosis and treatment strategies of tumors. The DCNN‐based pathological classification of tumor based on the SR‐PCT microscopic inner structural feature datasets has good potential in tumor staging diagnosis and clinical evaluation. The quantitative calculation and analysis based on machine learning of GLCM and GGCM can provide a data support and scientific evidence for tumorigenesis and its development.

## CONFLICTS OF INTEREST

No potential conflicts of interest were disclosed.

## AUTHORS' CONTRIBUTIONS

Acquisition and processing of experimental data and writing: Gong‐Xiang Wei and Yun‐Yan Liu; Pathological analysis and interpretation of tumorigenesis and progression: Xue‐Wen Ji, Qiao‐Xin Li, Yan Xing; Experimental technique support: Yan‐Ling Xue; Design, review, and revision of the manuscript: Hui‐Qiang Liu.

## Data Availability

The data used to support the findings of this study are available from the corresponding author upon request.
